# In Vivo Evaluation of the Biocompatibility of Surface Modified Hemodialysis Polysulfone Hollow Fibers in Rat

**DOI:** 10.1371/journal.pone.0025236

**Published:** 2011-10-25

**Authors:** Ganpat J. Dahe, Sachin S. Kadam, Siddharth S. Sabale, Dattatray P. Kadam, Laxman B. Sarkate, Jayesh R. Bellare

**Affiliations:** 1 Department of Chemical Engineering, Indian Institute of Technology Bombay, Powai, Mumbai, India; 2 Department of Veterinary Pathology, Bombay Veterinary College, Parel, Mumbai, India; 3 Department of Surgery & Radiology, Bombay Veterinary College, Parel, Mumbai, India; Biomedical Research Foundation of the Academy of Athens, Greece

## Abstract

Polysulfone (Psf) hollow fiber membranes (HFMs) have been widely used in blood purification but their biocompatibility remains a concern. To enhance their biocompatibility, Psf/TPGS (d-α-tocopheryl polyethylene glycol 1000 succinate) composite HFMs and 2-methacryloyloxyethyl phosphorylcholine (MPC) coated Psf HFMs have been prepared. They have been evaluated for in vivo biocompatibility and graft acceptance and compared with sham and commercial membranes by intra-peritoneal implantation in rats at day 7 and 21. Normal body weights, tissue formation and angiogenesis indicate acceptance of implants by the animals. Hematological observations show presence of post-surgical stress which subsides over time. Serum biochemistry results reveal normal organ function and elevated liver ALP levels at day 21. Histological studies exhibit fibroblast recruitment cells, angiogenesis and collagen deposition at the implant surface indicating new tissue formation. Immuno-histochemistry studies show non-activation of MHC molecules signifying biocompatibilty. Additionally, Psf/TPGS exhibit most favorable tissue response as compared with other HFMs making them the material of choice for HFM preparation for hemodialysis applications.

## Introduction

Membrane technology has proven vital in blood purification applications, especially hemodialysis. In hollow fiber membranes (HFMs), purification is achieved by regulating blood flow through the lumen as the dialysate flows counter-currently outside. The porous structure of the membrane facilitates diffusion of uremic toxins like urea, creatinine etc. from blood to dialysate without the loss of important blood proteins such as albumin. Desirable characteristics of such HFMs include high flux, selectivity and biocompatibility [1]. However, commercially available and most-widely used polysulfone (Psf) hemodialysis membranes have repeatedly been shown to be associated with clinical complications like hypersensitivity reactions, neutropenia, oxidative stress, contact and complement activation [2]–[5]. This translates into decreased quality of life, life expectancy and mortality of hemodialysis patients and has limited the success rates of such membranes [6], [7]. Thus, there is a need to enhance the biocompatibility of such membranes without compromising flux and selectivity.

Biocompatibility is the most desirable property of a biomaterial, of being biologically compatible by not producing a toxic, injurious and immunological response in living tissues or blood for the case of extra-corporeal devices [8]. The most commonly accepted mode of improving HFM biocompatibility has been modification of surface chemistry. Ishihara et al. achieved improved hemocompatibility of cellulose membranes under in vitro conditions by grafting with 2–methacryloyloxyethyl phosphorylcholine (MPC) polymers [9]. Polyacrylonitrile dialysis membranes, modified by covalent immobilization of chitosan/heparin polyelectrolyte complexes, exhibited improved antithrombogenicity and reduced platelet adhesion, thrombus formation and protein adsorption [10]. In our earlier studies on NIH3T3 cells, we showed that impregnation of d-α-tocopheryl polyethylene glycol 1000 succinate (TPGS) in Psf matrices enhances the biocompatibility of native HFMs [11]. However, definitive in vivo studies are required to assess the biocompatibility of such surface modified HFMs before their practical application in hemodialysis.

During a typical hemodialysis procedure, blood is circulated at 200 ml/min through HFMs (surface areas ∼1–2 m^2^) for 3–5 h thrice a week and such procedures lasts throughout the life of a renal failure patient [12]. The continuous, long-term exposure of blood to such membranes initiates various cellular reactions and protein conformational changes, depending on the physico-chemical nature of HFM surface. Hence, as per ISO 10993-1, systemic toxicity evaluation of membrane material for a week is necessary before hemodialysis trials [13]. These evaluations require studies on circulating blood for which number of animal models have been used [14]. Intra-peritoneal implantation in rats is a proven method for systemic evaluations, since it exposes the samples to both fluid and a variety of mesenchymal cell types of abdominal cavity [15].

In the present study, we have evaluated the in vivo biocompatibility of modified Psf/TPGS composite HFMs and inner-surface coated MPC/Psf HFMs on Wistar rat model. The effects of implanted membrane material on vital organ systems such as liver, kidney etc. have been studied by hematology, serum biochemistry and peritoneal fluid cytology. Tissue and immunological responses to HFMs have been evaluated using histopathological observations and immuno-histochemistry. These studies exhibit that Psf/TPGS membranes exhibit improved biocompatibility as compared to the other studied membranes and are suitable for practical hemodialysis applications.

## Results and Discussion

### Gross Observations

**Figure 1:**
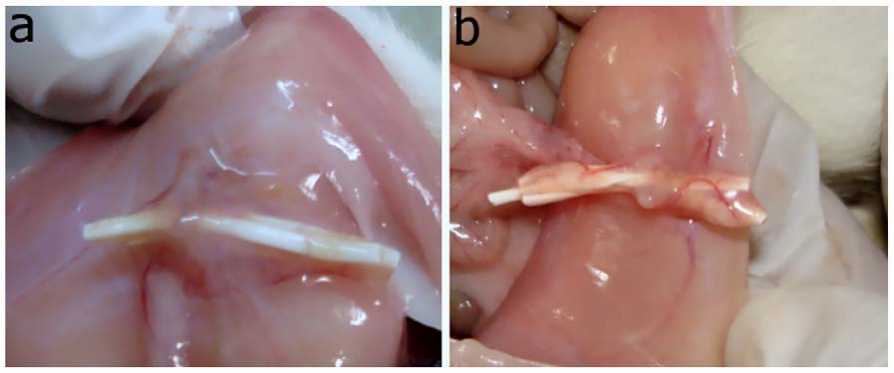
Interaperitoneal implantation of HFM. Images of Psf implants in peritoneum during excision in CO_2_ euthanized rat at days 7 (a) and days 27 (b).

**Figure 2:**
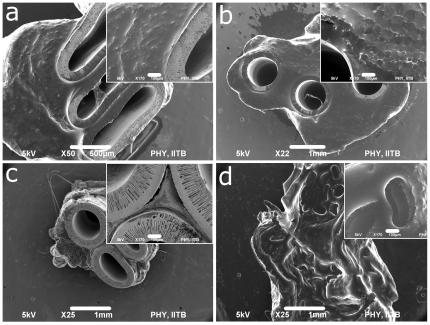
Tissue-HFM interaction study by Scanning Electron Microscopy. SEM micrographs of HFMs implants at day 21 (a) Psf (b) Psf/TPGS (c) MPC coated Psf and (d) Hemoflow F6 showing integration developed tissue with HFMs implants [scale bar: (a) 500 µm and (b), (c), (d) 1 mm (inset: (a), (b), (c) and (d) 100 µm)].

In any in vivo implant study the change in body weight is an essential parameter to judge health of animal model. The basal and final weights of rats at the time of implantation and after exposure are shown in Table 1. After HFM exposure for 7 days, the body weights of rats of normal, sham, Psf/TPGS and Hemoflow F6 groups were increased by 20–30 gm and for Psf and Psf/MPC groups, by 8–9 gm. By the 21st day, the increase in body weight was 40–50 gm across all groups indicating healthy condition of rats. Figure 1a and 2b are the camera images of Psf HFMs implants in peritoneal cavity on day 7 and 21, respectively. Both cases exhibit tissue formation on day 7 which becomes distinct and accompanied by angiogenesis on day 21.

**Table 1:** Basal and final weight of normal, sham surgeries and implanted rats.

	Day 7 (in gm)		Day 21 (in gm)	
Sample Type	Basal Weight	Final Weight	Basal Weight	Final Weight
Normal	150.00±14.14	169.50±30.60	170.50±30.40	220.50±3.53
Sham Surgeries	150.50±16.26	180.00±5.66	173.00±2.83	220.00±1.41
Psf	156.33±16.77	165.33±22.37	165.33±2.08	213.00±17.35
Psf/TPGS	165.67±15.95	189.33±8.33	154.33±9.07	191.00±9.90
Psf/MPC	164.00±10.15	172.33±0.58	159.67±13.65	206.33±17.62
Hemoflow F6	134.33±22.55	165.33±14.22	180.00±8.00	227.00±6.56

### Blood Hematology and Serum Biochemistry Study

Blood hematology studies are carried out to determine surgery-associated infections during implantation while serum biochemistry studies indicate functionalities of vital organs such as liver and kidney. CBCs, differential leukocyte counts and serum protein levels in blood of all groups after days 7 and 21 are listed in data S1. CBCs across all groups were within the normal range [20] after 7 and 21 days indicating that the implantation procedures were devoid of any infections.

Slightly increased neutrophil numbers in Leukocyte differential count on day 7 for Psf, Psf/TPGS, Psf/MPC and Hemoflow F6 groups indicated the presence of a mild inflammatory response as compared to control and normal groups. These numbers subsided and reached the normal level on the 21st day of implantation. This is a usual phenomenon, which occurs in response to foreign implants and is characterized by reduction in the inflammatory response with increase in the implantation period [21].

In serum biochemistry measurements (data S1), liver enzymes and serum proteins of all groups were within the normal range on days 7 and 21. However, as compared to day 7, the ALT, AST, and ALP enzyme concentrations for Psf, Psf/TPGS, Psf/MPC and Hemoflow F6 groups were elevated on day 21 and were even greater than the sham group. ALP has been documented as a possible marker for wound healing [22] and may also indicate tissue repair and angiogenesis. The observed ALP increase in implanted groups only may, thus, be due to these processes. The creatinine and blood urea nitrogen (BUN) values were within normal ranges for all groups indicating healthy and functional condition of kidney. Since all the implants are non-biodegradable and non-leachable, so no malfunction of liver and kidney was observed.

### Peritoneal Fluid Cytology

Differential leukocyte counts performed on peritoneal fluid collected on days 7 and 21 are listed in Table 2. As an initial response to HFM implants, increased polymorphonucleocyte (PMN) numbers were observed in the peritoneal fluid at day 7 when compared to the sham and normal groups. However at day 21, these numbers were within the normal range. The elevated numbers may be due to the surgical procedure. Similar trends were observed for neutrophils in differential leukocyte counts, indicating the presence of post-surgical stress which subsides with time. The decrease in this stress occurs because of wound healing and tissue formation as the time progresses.

**Table 2:** Leukocyte differential counts for peritoneal fluid after HFM implantation.

Sample Type	Incubation Time (days)	PMN*	Mononuclear**	Lymphocytes	Eosinophils	Basophils
Normal (Without Surgery)	7	21±2	61±6	16±3	1±0	1±0
Sham Surgeries	7	30±1	51±2	15±2	1±0	1±0
Psf	7	42±3	40±2	15±3	2±1	1±0
Psf/TPGS	7	39±3	41±1	17±3	2±1	1±0
Psf/MPC	7	42±4	41±4	14±0	2±1	1±0
Hemoflow F6	7	39±3	43±4	15±2	2±1	1±0
Normal (Without Surgery)	21	19±2	61±4	18±3	1±0	1±0
Sham Surgeries	21	20±1	66±2	11±2	2±1	1±0
Psf	21	21±2	61±5	15±2	2±0	1±0
Psf/TPGS	21	25±2	57±4	16±3	2±1	0±0
Psf/MPC	21	24±2	60±2	13±3	2±1	1±0
Hemoflow F6	21	19±2	65±4	15±2	1±0	0±0

(*PMN = polymorph nucleocytes, i.e. granulocytes; **Mononuclear = monocytes, macrophages, and mesothelial).

### Tissue-HFM interaction study by Scanning Electron Microscopy

SEM provides high resolution direct information on tissue-biomaterial interactions with minimal sample preparation as compared to histology. Further, histological sample preparation causes implant detachment from tissues and this information is preserved during SEM sample preparation. The SEM micrographs of HFM implanted groups at day 21 are shown in Figure 2. Dense tissue integration with the implant was observed in all the cases (see inset micrographs). No masses were observed in the implant bores or lumens. Degradation and material surface changes due to body fluids were not observed. However, it is difficult to extract detailed information about surrounding tissue/cells type from these micrographs because of surface similarities in different types of cells.

### Histological Study of Implants

**Figure 3:**
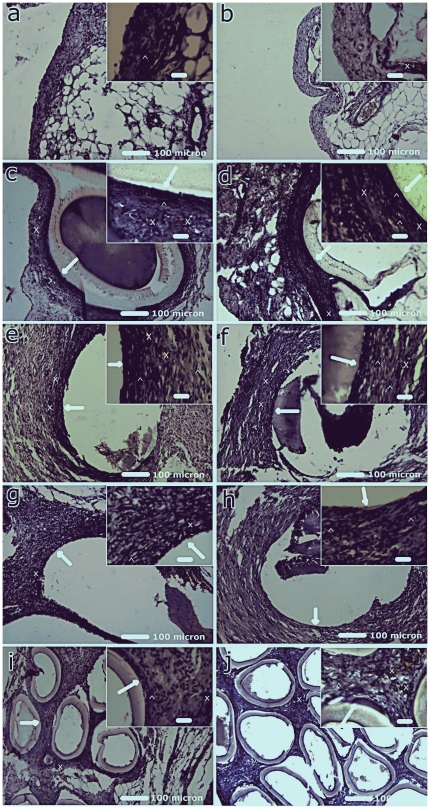
Histopathological analysis of implant by hematoxylin and eosin stain. Optical photomicrograph of hematoxylin and eosin stained tissue section of normal rat peritoneum at day 7 (a) and day 21 (b); Psf HFMs implants at day 7 (c) and day (d); Psf/TPGS HFMs implants at day 7 (e) and day 21 (f); Psf/MPC HFMs implants at day 7 (g) and day 21(h); Hemoflow F6 HFMs implants at day 7 (i) and day 21 (j). Arrow head, cross and big white arrow denotes fibroblast, angiogenesis and interface of implant with tissue respectively. [Scale bar: 100 µm (inset: 20 µm)]. Note: Psf/TPGS (e and f) and Psf/MPC (g and h) implants were detached during tissue processing for histology, but tissue interface with implants are distinctly visible.

The optical microscopy based histological evaluation of tissues surrounding the implant provides morphological and pathological analysis. Tissue reactions in the implanted groups are summarized in Table 3 and optical micrographs of respective histological sections at day 7 and day 21 are shown in Figure 3. On day 7 post-implantation, a relatively greater infiltration of polymorphonuclears (PMNs) and macrophages into the surrounding tissues of MPC and Hemoflow F6 implants was observed as compared to the Psf and Psf/TPGS implants. However, infiltration of fibroblasts surrounding the implant and degrees of angiogenesis were more prominent for Psf and Psf/TPGS implants than Psf/MPC and Hemoflow F6 implants. On day 21, fibroblast recruitment was enhanced, while angiogenesis remained the same for all cases as compared to day 7. PMN, macrophage and lymphocyte infiltrations were reduced on day 21 as compared to day 7. Similar trends of reduction in PMNs, macrophages and lymphocytes with time upon implantation of disulfide-crosslinked hyaluronan films have been reported earlier [23].

**Table 3:** Inflammatory evaluation of intraperitoneal HFMs implants.

Implantation Period (days)	Implant Type	PMC	Macrophages	Lymphocytes	Fibroblast	Angiogenesis	Collagen Bundles
7	Psf	++	+	+	+	++	Thin
21	Psf	+	+	+	++	++	Thick
7	Psf/TPGS	+	+	+	++	++	Thin
21	Psf/TPGS	+	++	+	+++	++	Thick
7	Psf/MPC	+	+	++	++	++	Thin
21	Psf/MPC	+	++	+	+++	++	Thick
7	Hemoflow F6	++	+	++	+	+	Thin
21	Hemoflow F6	+	++	+	++	++	Thick

**Figure 4:**
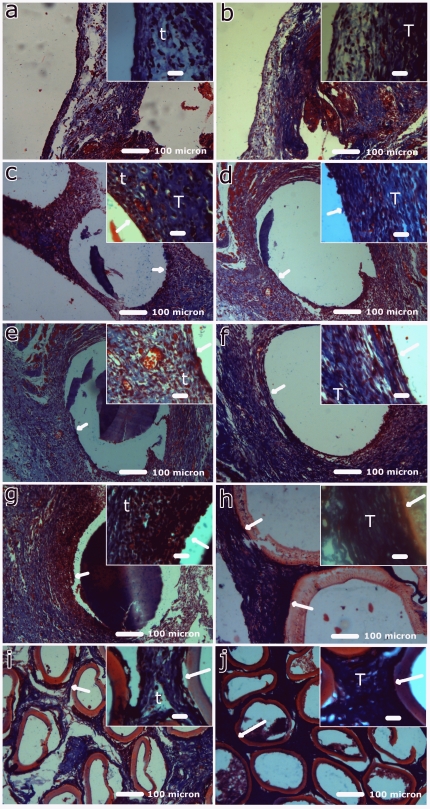
Histopathological analysis of implant by Masson's trichrome stain. Optical photomicrograph of Masson's trichrome stained tissue section of normal normal rat peritoneum at day 7 (a) and day 21 (b); Psf HFMs implants at day 7 (c) and day 21 (d); Psf/TPGS HFMs implants at day 7 (e) and day 21 (f); Psf/MPC HFMs implants at day 7 (g) and day 21 (h); Hemoflow F6 HFMs implants at day 7 (i) and day 21 (j). The symbols t and T denotes thin, thick blue collagen bundles respectively, while arrow shows interface implant with tissue. [Scale bar: 100 µm (inset: 20 µm)]. Note: Some hollow fiber implants are detached (c, d, e, f, and g) during processing for histology.

Collagen deposition is vital for new tissue formation and its distribution and extent can be studied by Masson's trichrome staining. Figure 4 shows the optical micrographs of Masson's trichrome stained sections of different groups at days 7 and 21. On day 7 post-implantation, thin collagen bundles (t) were observed for all implants, while thick collagen bundles (T) were observed on day 21. The increased collagen deposition may be due to increase in fibroblast numbers (Table 3) which secrete collagen and is an indicator of tissue formation. It has also been reported earlier for poly(l-lactide-co-e-caprolactone) scaffolds implanted subcutaneousely [24].

These histological studies exhibit favorable tissue response to all implants indicating biocompatibility. However, the Psf/TPGS group showed minimum lymphocytes and maximum fibroblasts and angiogenesis (Table 3). These observations indicate that Psf/TPGS achieves better tissue response as compared other HFM implants, including commercial HFMs. This may be due to the presence of TPGS, which leaches and cleaves into vitamin E and polyethylene glycol moieties by enzymatic reactions [25]. Vitamin E, a proven anti-oxidant [26], causes a reduction in implant-associated oxidative stress and contributes to the better performance of the Psf/TPGS implant.

### Immunohistochemistry by Confocal Microscopy

**Figure 5:**
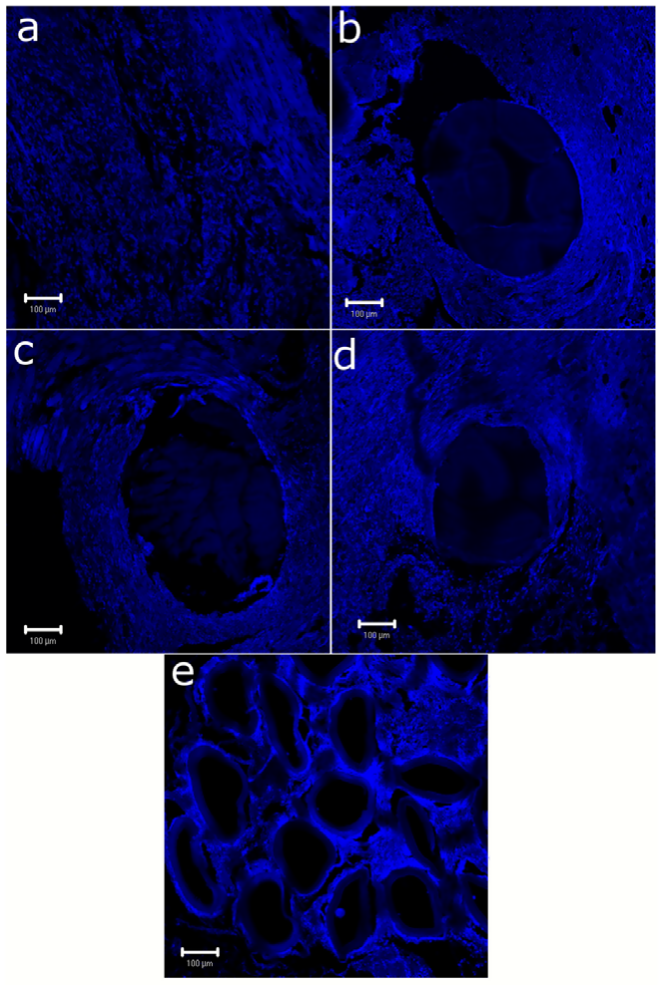
Immunohistochemistry of implants. Confocal laser micrographs of immunohistochemical stained tissue of (a) Psf HFMs, (b) Psf/TPGS HFMs, (c) Psf/MPC HFMs and (d) Hemoflow F6 HFMs implants with DAPI for nuclei (blue) [scale: 100 µm].

Studies of immunological response of tissues with HFM implants are vital for assessing host-verses-graft (implant) reactions. HLA-DR is constitutively expressed on antigen-presenting immune system cells like dendritic cells, B cells, and monocytes/macrophages and its expression is further up-regulated upon activation. It is, thus, considered as an essential marker for activation of immune system [27], [28]. The confocal micrographs of normal peritoneal tissue and implanted groups stained with DAPI for nuclei and phycoerythrin (PE) conjugated HLA- DR antibody for MHC at day 21 are shown in Figure 5. The micrographs showed distinct nuclei of cells surrounding the implants; however, fluorescence due to PE was not observed indicating non-activation of immune cells in the tissues surrounding the implants. Also, peritoneal fluid cytology showed normal leukocyte differential count in implanted rats. This supports non-activation of immune system at the implant site and indicates acceptance of the implants by the animals.

Psf-based implants with different surface charcateristics were evaluated for their in vivo biocompatibility in rat model. These implants exhibited improved biocompatibility over commercially available membranes. The post-implantation CBC, renal and liver function tests indicated normal health of rat signifying absence of infections due to surgical procedures. Peritoneal fluid cytology exhibited elevated PMNs at day 7 post-implantation due to initial inflammation which returned within the normal range by day 21 indicating absence of chronic inflammation. Histopathology studies revealed abundunt fibroblast and angiogenesis in the tissues surrounding Psf/TPGS implants as compared to Psf, Psf/MPC and Hemoflow F6 implants indicating imporved biocompatiblity of Psf/TPGS implants attributable to the cleaved Vitamin E moiety. Immune responses against all the implants were absent. This study may be useful for generation of hollow fiber based vascular grafts which are able to grow within the peritoneal cavity.

## Materials and Methods

### Ethics Statement

All the animal experimental protocols were approved by Committee for the Purpose of Control and Supervision of Experiments on Animals (CPCSEA), India and animal ethical committee of Mumbai Veterinary College (MVC), Mumbai.

### Preparation of Hollow Fiber membrane

Psf (UDELTM P-3500 LCD MB7-BULK) was procured from M/s. Solvay Advanced Polymers, USA and dried in vacuum oven for 24 h at 90°C to remove absorbed water. Vitamin E TPGS (NF grade) and MPC polymers PC 2118 [Poly(2-methacryloyloxyethyl)-2′-(trimethylammoniumethyl) phosphate, inner salt)-co-(3-(trimethoxysilyl)propyl methacrylate)-co-(hydroxypropyl methacrylate)] were generously gifted by M/s. Isochem SA (Paris, France) and M/s. Vertellus Specialties Inc., (Basinstoke, UK), respectively. The solvent, N-methyl2-pyrrolidone (NMP), was procured from S.D. Fine-Chem Ltd., India. Psf and Psf/TPGS composite HFMs were prepared as per the conditions and compositions listed in Table 4. The prepared fibers were kept in water for one day to remove the residual solvent and then used for further studies.

**Table 4:** Process parameters used for hollow fiber membrane preparation.

Parameters	Psf HFMs	Psf/TPGS HFMs	MPC coated Psf HFMs (Psf/MPC)
Ambient Temperature (°C)	25	25	25
Relative Humidity (%)	50–60	50–60	50–60
Dope Solution Composition	Psf/NMP (25/75)	Psf/TPGS/NMP (25/20/55)	Psf/NMP (25/75)
Bore Solution Composition	DI Water	DI Water	10 mg/ml PC 2118 in DI water
Dope Solution Temperature (°C)	25	25	25
Bore Solution Temperature (°C)	25	25	25
Dope Flow Rate (ml/min)	2	2	2
Bore Flow Rate (ml/min)	2.5	2.5	2.5
Spinneret ID/OD (mm)	0.8/1.4	0.8/1.4	0.8/1.4
Air Gap (cm)	45	45	45
Coagulation Bath Composition	RO Water	RO Water	RO Water
Rinse Bath Composition	RO Water	RO Water	RO Water
Coagulation Bath Temperature (°C)	25	25	25
Rinse Bath Temperature (°C)	35	35	35
Take-up Drum Velocity (m/min)	3.89	3.01	3.89

### Implantation of HFM into Peritoneal Cavity

Wistar female rats (age group of 4–5 weeks) were procured from Bombay Veterinary College animal house and were housed under controlled conditions of light (12 h light and 12 h darkness), temperature (24°C) and humidity (50%) and maintained on normal chow and water.

Recipient rats were anesthetized (ketamine-80 mg/kg and xylazine-8 mg/kg, i.p.), shaved, and cleaned and were subjected to a laparotomy through a 1 cm long incision on the lower right abdominal wall. The exposed area was kept moist with normal saline swab. A bunch of three HFMs (each 1.5 cm long) was slowly delivered inside the peritoneal cavity. Finally, the peritoneum and skin were sutured using absorbable 6–0 catgut sutures (Stericat Gutstrings, Delhi). Approximately 2 mm part of the HFMs was kept above the peritoneum while suturing to avoid implant dislocation.

The study consisted of 6 groups (n = 3) corresponding to Psf, Psf/TPGS, Psf/MPC HFMs and commercial membranes (Hemoflow F6, Fresenius Medical Care), normal (no surgery) and sham (surgery without implant). Samples were assessed on day 7 and day 21. All rats (control and experimental) received an i.p. injection of gentamycin (3 mg/kg body weight), ampicillin and cloxacillin (20 mg/kg body weight) and diclofenac sodium (0.5 mg/kg body weight) for 3 days (starting from the day of operation) in addition to the topical ointments (Soframycin®, Aventis Pharma. Ltd., Pune, India) and placed in a cage on a heating pad.

### Hematology and Serum Biochemistry of transplanted rats

A complete blood count was performed for diagnosis of infections and inflammatory responses. Retro-orbital blood collection was performed in two vials (with and without heparin, 1 ml each) as described by Sorg and Buckner [16] on days 7 and 21. The heparin (2 IU/ml) containing vial was used for hematological studies, while second vial without heparin was used for serum biochemistry study. Complete Blood Count (CBC) was performed using Abacus (Diatron MI PLC, Hungary) hematology analyzer, while serum biochemistry evaluation was done on Erba Chem 7 (Erba Mannheim, Germany) semi-autoanalyzer using commercial reagent kits.

### Peritoneal Fluid Cytology

Peritoneal fluid cytology was carried out for evaluating inflammatory responses due to implantation. On days 7 and 21, three rats from each group were scarified by euthanizing in a CO_2_ chamber and their abdominal cavities were exposed. The cavities were filled with 5 ml of chilled phosphate buffer saline (PBS) and were massaged gently for 3–5 min. The PBS solution was aspirated; cytospinned and stained using Wright-Giemsa stain (Sigma Aldrich, MO, USA). The slides were then air-dried and a leukocyte differential count was performed by counting the cells in a standard clinical hemocytometer.

### Histological Study of Implants

Histopathological evaluation was carried out on days 7 and 21 for sacrificed rats from all the different groups. HFM implants along with the surrounding tissue were excised and fixed with 10% formalin, embedded in paraffin, sectioned (2–3 µm thick) with a microtome at three different distances from the surface, and stained with hematoxylin and eosin (H&E) (Sigma Aldrich, MO, USA) as per standard protocol [17]. Sections were then examined for the presence of fibrin, exudates, induction of vascularization, and formation of fibrous capsule. Sections were also stained with Masson's Trichrome and observed for extent and distribution of collagen fibers in tissue [18].

### Scanning electron microscopy (SEM) Study

Fixed HFM implants with tissue were sectioned with a sharp cutter, dehydrated with graded alcohol and dried at room temperature. These samples were coated with gold/palladium using SC7640 Sputter Coater (Quorum Technologies Ltd, UK) and observed under scanning electron microscope (Hitachi, S-3400 N, UK).

### Immunohistochemistry Study

Implant sections were prepared as described above. Paraffin was removed using xylene and the sections were hydrated using a series of washes in graded alcohol. The sections were then washed with PBS and heated in 10 mM sodium citrate using microwave oven for antigen retrieval. They were fixed with 4% paraformaldehyde for 20 min, washed with PBS and permeabilized using 0.1% triton-x solution [19]. This was followed by exposure to phycoerythrin (PE) conjugated HLA- DR antibody (Santa Cruz Biotechnology, Inc., CA, USA) (1∶200) for 1 h in dark at room temperature to stain major histocompatibility complex (MHC) antigens. Finally, sections on glass slides were mounted using an antifade-containing mounting medium (Vectashield, Vector laboratories, Burlingame, USA) and 4′,6-diamidoino-2-phenylindole (DAPI) (Sigma-Aldrich, USA). Images were captured using a Zeiss-LSM 510 laser scanning confocal microscope (Carl Zeiss Meditec AG, Jena, Germany) using 10× objectives.

## Supporting Information

Data S1 **Hematology and serum biochemistry of blood collected on day 7 & 21.**
(DOC)
