# Supplementary material for: In Vivo Evaluation of the Biocompatibility of Surface Modified Hemodialysis Polysulfone Hollow Fibers in Rat
Source: PLoS One. 2011 Oct 25;6(10):e25236. doi: 10.1371/journal.pone.0025236 (PMC3201955; doi:10.1371/journal.pone.0025236)
Supplement: Data S1 — Hematology and serum biochemistry of blood collected on day 7 & 21. (DOC) [file pone.0025236.s001.doc]

Supplementary table: Hematology and serum biochemistry of blood collected on day 7.

| Sample Type/Parameters | Normal Range [170,173,174] | Normal Without Surgery | Sham Surgery | Psf | Psf/TPGS | Psf/MPC | Hemoflow F6 |
| --- | --- | --- | --- | --- | --- | --- | --- |
| **Complete Blood Count** | |  |  |  |  |  |  |
| Hb (g/dl) | 11-19.2 | 16.65±0.21 | 14.25±0.35 | 18.73±0.71 | 16.47±1.62 | 19.70±1.99 | 14.00±0.70 |
| PCV (%) | 36-54 | 55.65±0.49 | 46.35±0.49 | 63.43±2.44 | 52.90±3.81 | 67.73±4.16 | 48.33±3.52 |
| MCV (fl) | 48-70 | 57.00±1.41 | 59.00±0.00 | 53.67±3.06 | 57.67±3.04 | 57.00±1.73 | 56.00±3.00 |
| MCH (pg) | 17.8-20.8 | 17.10±0.00 | 18.15±0.21 | 15.80±0.72 | 17.80±1.12 | 16.63±0.58 | 16.20±1.32 |
| MCHC (%) | 30-34 | 30.30±0.14 | 30.70±0.42 | 29.53±0.85 | 30.80±1.44 | 29.13±0.90 | 29.00±1.11 |
| Platelets(10^3^/μL) | 500-1300 | 955.00±78.38 | 601.50±73.45 | 625.00±68.14 | 448.00±84.31 | 710.33±62.48 | 483.00±34.14 |
| WBC (10^3^/μL) | 6-15 | 10.65±0.21 | 9.66±1.75 | 6.05±3.11 | 11.77±3.17 | 9.56±2.49 | 14.80±1.49 |
| RBC (10^6^/μL) | 7-12.5 | 9.91±0.07 | 7.85±0.12 | 11.87±1.03 | 9.15±0.92 | 11.83±1.59 | 8.68±0.74 |
| **Differential Leukocyte Count** | |  |  |  |  |  |  |
| Neutrophils (%) | 9-34 | 19.00±1.41 | 19.50±0.71 | 31.67±4.71 | 40.33±3.65 | 44.67±1.68 | 25.67±1.53 |
| Lymphocytes (%) | 65-85 | 77.00±1.41 | 76.50±0.71 | 63.33±7.71 | 57.00±4.11 | 52.00±3.89 | 71.67±1.15 |
| Monocytes (%) | 0-5 | 1.50±0.71 | 3.00±1.41 | 3.67±0.58 | 1.33±0.58 | 2.00±0.00 | 1.33±0.58 |
| Eosinophils (%) | 0-6 | 2.50±0.71 | 1.00±0.00 | 1.33±0.58 | 1.33±0.58 | 2.00±1.73 | 1.33±0.58 |
| Basophils (%) | 0-1.5 | 0.00±0.00 | 0.00±0.00 | 0.00±0.00 | 0.00±0.00 | 0.00±0.00 | 0.00±0.00 |
| **Liver Function Test** | |  |  |  |  |  |  |
| ALT IU/L | 35-80 | 65.30±5.73 | 66.76±0.59 | 33.86±5.66 | 57.04±2.78 | 44.21±6.68 | 49.13±4.01 |
| AST IU/L | 65-203 | 53.50±1.70 | 52.45±4.45 | 69.29±3.96 | 72.13±5.92 | 77.33±2.94 | 68.97±3.22 |
| ALP IU/L | 26-147 | 60.75±0.78 | 45.60±4.67 | 72.63±3.29 | 55.03±4.18 | 66.27±3.20 | 64.13±4.20 |
| Protein (g/dl) | 5.6-7.6 | 6.37±0.02 | 7.07±0.33 | 6.05±0.08 | 6.73±0.31 | 7.66±0.17 | 7.23±0.11 |
| Albumin (g/dl) | 2.8-4.8 | 3.28±0.18 | 3.47±0.06 | 3.32±0.31 | 3.72±0.29 | 4.00±0.14 | 3.50±0.07 |
| Globulin (g/dl) | 3.4-4.8 | 3.09±0.16 | 3.61±0.26 | 2.73±0.26 | 3.01±0.59 | 3.66±0.13 | 3.73±0.17 |
| **Renal Function Test** | |  |  |  |  |  |  |
| Creatinine (mg/dl) | 0.2-0.8 | 0.47±0.17 | 0.47±0.10 | 1.07±0.11 | 1.14±0.05 | 1.11±0.13 | 0.84±0.16 |
| BUN (mg/dl) | 10-21 | 16.80±0.71 | 21.75±1.91 | 16.97±5.86 | 16.07±1.97 | 18.47±2.25 | 20.40±1.01 |

(Hb=Hemoglobin, PCV=packed cell volume, MCV=mean corpuscular volume, MCH=mean corpuscular hemoglobin, MCHC=mean corpuscular hemoglobin concentration, WBC=white blood cells, RBC=red blood cells, ALT= alanine aminotransferase, AST=aspartate aminotransferase, ALP=alkaline phosphatase, BUN=blood urea nitrogen)

Supplementary table: Hematology and serum biochemistry of blood collected on day 21.

| Sample Type/Parameters | Normal Range [170,173,174] | Normal Without Surgery | Sham Surgery | Psf | Psf/TPGS | MPC | Hemoflow F6 |
| --- | --- | --- | --- | --- | --- | --- | --- |
| **Complete Blood Count** | |  |  |  |  |  |  |
| Hb (g/dl) | 11-19.2 | 15.85±0.21 | 15.30±0.85 | 16.67±1.40 | 15.10±0.69 | 14.27±0.46 | 14.23±1.34 |
| PCV (%) | 36-54 | 51.20±1.13 | 49.95±2.33 | 54.37±4.74 | 49.10±2.01 | 46.43±1.10 | 45.53±4.36 |
| MCV (fl) | 48-70 | 55.95±2.90 | 55.00±2.83 | 55.67±2.31 | 60.67±5.69 | 57.67±8.08 | 59.00±4.36 |
| MCH (pg) | 17.8-20.8 | 17.25±0.92 | 16.50±0.71 | 17.13±0.64 | 18.63±1.59 | 18.03±2.31 | 18.40±1.48 |
| MCHC (%) | 30-34 | 30.95±0.35 | 30.20±0.28 | 30.73±0.21 | 30.77±0.21 | 30.73±0.23 | 31.20±0.26 |
| Platelets(10^3^/μL) | 500-1300 | 608.00±48.08 | 563.00±32.53 | 487.67±43.15 | 530.33±24.58 | 598.67±75.19 | 550.67±54.50 |
| WBC (10^3^/μL) | 6-15 | 13.15±0.64 | 14.15±1.18 | 15.97±5.29 | 12.20±5.48 | 11.43±0.35 | 14.03±6.20 |
| RBC (10^6^/μL) | 7-12.5 | 9.19±0.40 | 9.11±0.13 | 9.76±1.07 | 8.16±1.02 | 7.70±0.88 | 7.81±1.25 |
| **Differential Leukocyte Count** | |  |  |  |  |  |  |
| Neutrophils (%) | 9-34 | 16.00±1.41 | 19.00±4.24 | 15.00±1.00 | 19.67±8.14 | 25.33±5.51 | 15.67±1.53 |
| Lymphocytes (%) | 65-85 | 82.00±1.41 | 77.50±2.12 | 82.67±2.08 | 79.33±8.14 | 71.33±6.66 | 79.33±2.31 |
| Monocytes (%) | 0-5 | 1.00±0.00 | 2.00±1.41 | 1.33±0.58 | 1.00±0.00 | 2.00±1.00 | 3.00±1.00 |
| Eosinophils (%) | 0-6 | 1.00±0.00 | 1.50±0.71 | 1.00±0.00 | 0.00±0.00 | 1.33±0.58 | 2.00±0.00 |
| Basophils (%) | 0-1.5 | 0.00±0.00 | 0.00±0.00 | 0.00±0.00 | 0.00±0.00 | 0.00±0.00 | 0.00±0.00 |
| **Liver Function Test** | |  |  |  |  |  |  |
| ALT IU/L | 35-80 | 51.14±7.30 | 66.95±3.46 | 62.74±7.60 | 60.08±4.31 | 87.15±5.66 | 72.00±2.59 |
| AST IU/L | 65-203 | 102.10±10.61 | 91.30±7.07 | 166.03±25.64 | 129.53±12.50 | 191.90±25.03 | 138.37±24.23 |
| ALP IU/L | 26-147 | 67.13±8.95 | 55.30±1.41 | 104.83±19.86 | 113.30±9.64 | 122.07±22.03 | 143.57±9.50 |
| Protein (g/dl) | 5.6-7.6 | 8.79±3.45 | 6.94±0.04 | 7.35±0.26 | 7.57±0.38 | 7.69±0.15 | 7.51±0.32 |
| Albumin (g/dl) | 2.8-4.8 | 3.65±0.70 | 3.52±0.08 | 3.53±0.40 | 3.80±0.10 | 3.65±0.23 | 3.35±0.30 |
| Globulin (g/dl) | 3.4-4.8 | 4.15±2.75 | 3.43±0.12 | 3.82±0.19 | 3.77±0.43 | 4.04±0.15 | 4.16±0.45 |
| **Renal Function Test** | |  |  |  |  |  |  |
| Creatinine (mg/dl) | 0.2-0.8 | 0.59±0.34 | 0.47±0.30 | 1.01±0.12 | 1.03±0.33 | 0.93±0.19 | 0.94±0.05 |
| BUN | 15-21 | 16.19±2.67 | 16.80±0.71 | 19.00±4.18 | 19.33±1.13 | 22.94±1.47 | 17.84±1.50 |
